# Crystal Structure of Bright Fluorescent Protein BrUSLEE with Subnanosecond Fluorescence Lifetime; Electric and Dynamic Properties

**DOI:** 10.3390/ijms24076403

**Published:** 2023-03-29

**Authors:** Ekaterina Goryacheva, Roman Efremov, Nikolai Krylov, Igor Artemyev, Alexey Bogdanov, Anastasia Mamontova, Sergei Pletnev, Nadya Pletneva, Vladimir Pletnev

**Affiliations:** 1 Shemyakin-Ovchinnikov Institute of Bioorganic Chemistry, Russian Academy of Sciences, Ulitsa Miklukho-Maklaya, 16/10, 117997 Moscow, Russiaefremov@nmr.ru (R.E.); krylovna@gmail.com (N.K.); artem1@ibch.ru (I.A.); noobissat@yandex.ru (A.B.); nadin.plet@gmail.com (N.P.); 2 Faculty of Applied Mathematics, National Research University Higher School of Economics, 101000 Moscow, Russia; 3 Moscow Institute of Physics and Technology (State University), 141701 Dolgoprudny, Russia; 4 Vaccine Research Center, National Institute of Allergy and Infectious Diseases, NIH, Bethesda, MD 20892, USA; sergei.pletnev2@nih.gov

**Keywords:** fluorescent protein, fluorescence lifetime, X-ray structure, molecular dynamics, computer modeling

## Abstract

The rapid development of new microscopy techniques for cell biology has exposed the need for genetically encoded fluorescent tags with special properties. Fluorescent biomarkers of the same color and spectral range and different fluorescent lifetimes (FLs) became useful for fluorescent lifetime image microscopy (FLIM). One such tag, the green fluorescent protein BrUSLEE (Bright Ultimately Short Lifetime Enhanced Emitter), having an extremely short subnanosecond component of fluorescence lifetime (FL~0.66 ns) and exceptional fluorescence brightness, was designed for FLIM experiments. Here, we present the X-ray structure and discuss the structure-functional relations of BrUSLEE. Its development from the EGFP (enhanced green fluorescent proteins) precursor (FL~2.83 ns) resulted in a change of the chromophore microenvironment due to a significant alteration in the side chain conformations. To get further insight into molecular details explaining the observed differences in the photophysical properties of these proteins, we studied their structural, dynamic, and electric properties by all-atom molecular-dynamics simulations in an aqueous solution. It has been shown that compared to BrUSLEE, the mobility of the chromophore in the EGFP is noticeably limited by nonbonded interactions (mainly H-bonds) with the neighboring residues.

## 1. Introduction

The GFP-like fluorescent proteins (FPs) have become essential noninvasive tools for visualization and monitoring of the biochemical processes within cells or whole organisms, and the range of their application is expanding continuously [1,2,3,4,5]. In the past decade, advances in fluorescence lifetime imaging grew from fundamental biological studies to advanced clinical diagnostics. Fluorescence lifetime-imaging microscopy (FLIM) is now effectively used in cell biology to monitor dynamic signaling events in the living cell [6,7]. It became an important research tool that provides for a new way to detect, visualize, and investigate the structure and function of biological systems, especially in the studies of protein–protein interactions using fluorescence resonance energy transfer (FRET) [8]. The fluorescence lifetime (FL) of the fluorophore, rather than its intensity, is used to create an image in the FLIM in time-resolved spectroscopy, demonstrating high sensitivity to the local microenvironment of the fluorophore. It produces spatially resolved images providing another dimension of information for visualizing fluorophores. FLIM can separate probes of the same color but different FL, providing an approach for multiparameter imaging.

Most GFP-like FPs available to date have a narrow range of FLs of 2.3–3.5 ns, which limits their application in a multiparameter FLIM. FPs with subnanosecond lifetimes remain virtually unexplored. The main issue of the method is that the shortening of the protein FL correlates with a decrease in its fluorescence quantum yield (FQY), and hence brightness. Until recently, green FPs with subnanosecond lifetimes were represented by variants with low-fluorescence brightness, which complicated their application in multiparameter FLIM.

**Figure 1:**
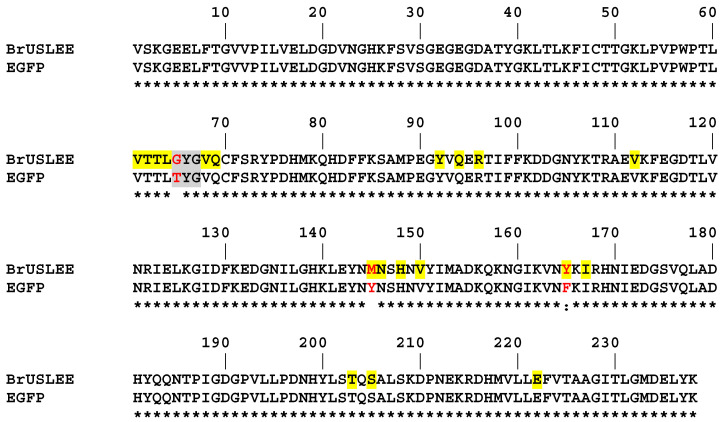
Alignment of the amino-acid sequences of BrUSLEE and its precursor EGFP. Differences are shown in red. The chromophore-forming triad is highlighted in gray. Residues from the chromophore nearest environment (<4 Å), which stabilize the chromophore by H-bonds (direct or via water) or hydrophobic interactions, are highlighted in yellow. The identical residues are marked with *, and the similar ones with:.

BrUSLEE (Bright Ultimately Short Lifetime Enhanced Emitter) is a new green FP probe (λex/ λem 487/509 nm) with two reliably detected time-resolved FL components: a short one with a subnanosecond FL and a τ of ~0.66 ns, and a long one with a τ~1.5 ns (Table 1) [9,10]. The protein was designed from EGFP by introducing three critical mutations, namely Thr65Gly, Tyr145Met, and Phe165Tyr (Figure 1). The first two mutations sharply decreased the FL, and the third increased the FQY. BrUsLEE shows an exceptional fluorescence brightness of the short FL component, reaching ~78% of that of the green EGFP precursor. The BrUsLEE demonstrates high performance in the multiparameter FLIM experiments and allows both a reliable detection of the probe and the recording of distinct FL signals in the presence of spectrally similar GFPs. Here, we present the X-ray structure of BrUSLEE and supporting results from the structure-based molecular dynamics (MD) simulation.

## 2. Results and Discussion

### 2.1. Overall Structure

The crystal asymmetric unit of BrUSLEE contains three dimers AC, BD, and EF. Each dimer comprises two monomers related by a noncrystallographic twofold symmetry axis with a side-to-side packing at ~120°. The interface between the dimer subunits has a contact area of ~2900 Å^2^. It is stabilized by nine direct hydrogen bonds and the hydrophobic cluster of six residues (see Table 2 for an example of EF interface). The principal structural fold of the BrUSLEE monomer, shared with all other members of the GFP family, is an 11-stranded β-barrel with loop caps from both sides.

### 2.2. Structural Features of the Chromophore Area

**Figure 2:**
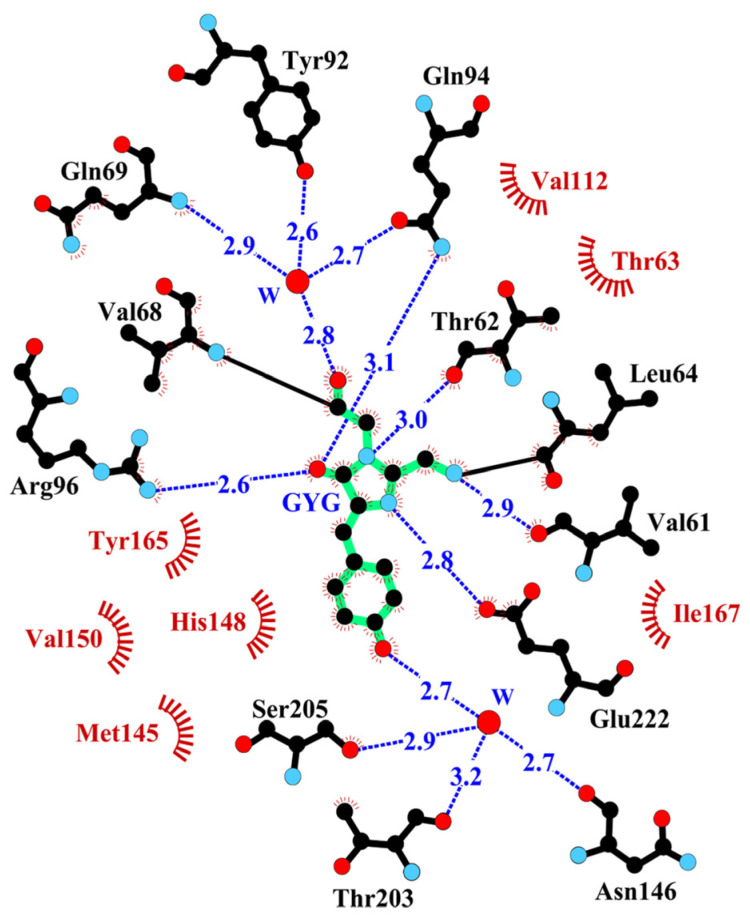
The nearest amino acid environment of the chromophore (shown in green) in the BrUSLEE structure within 4 Å. Hydrogen bonds (≤3.3 Å) are shown as blue dashed lines, water molecules (W) as red spheres, and van der Waals contacts as orange “eyelashes” (figure prepared with LIGPLOT/HBPLUS [11]).

**Figure 3:**
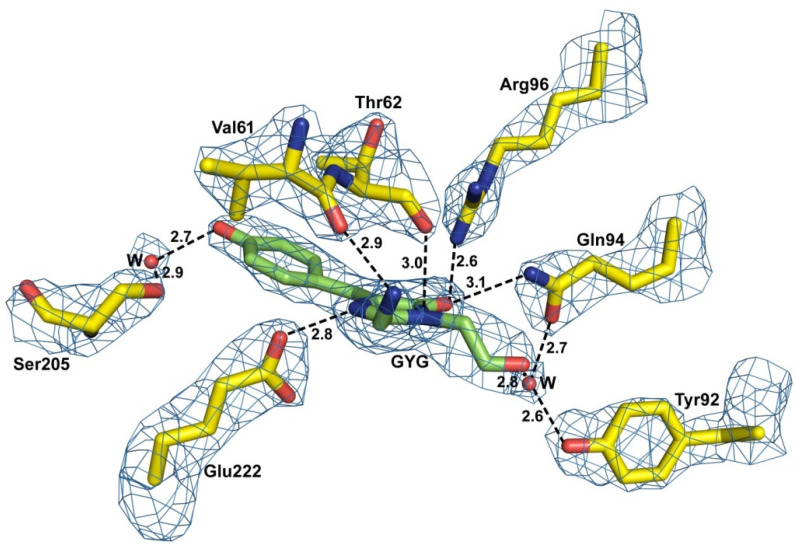
View of the GYG chromophore (shown in green) with key adjacent residues in the 2Fo-Fc electron density (cutoff *ρ* = 2.0 *σ*) Hydrogen bonds (≤3.3 Å) are shown as black dashed lines and water molecules (W) as red spheres (figure created with PYMOL [12]).

The principal structural fold of the BrUSLEE monomer, shared with all other members of the GFP family, is an 11-stranded β-barrel with loop caps from both sides. The nearest shell of the Gly65-Tyr66-Gly67 chromophore is composed of 19 residues, forming an extensive H-bond network around the chromophore (Figure 2 and Figure 3). The position of the chromophore is stabilized by direct H-bonds with five amino-acid residues and two water molecules mediating interaction with six other residues. Seven hydrophobic residues provide additional chromophore stabilization through the hydrophobic interactions.

Three mutations in the EGFP chromophore and its nearest environment (Thr65Gly, Tyr145Met, and Phe165Tyr) resulted in BrUSLEE. The substitutions increased the free volume around the chromophore, causing a 0.7 Å shift of the hydroxyl group of Tyr66. The mutation of the chromophore Thr65 to Gly shifted the H-binding of catalytic Glu222 from Thr65 to the imidazolinone nitrogen, and the replacement of Phe165 with Tyr led to the formation of an H-bonded chain–Tyr165∙∙∙∙Arg96∙∙∙∙O=C-imidazolinone. The replacement of Tyr145 by a relatively flexible met facilitated the conformational change of Thr203, accompanied by a disruption of its H-bond with the Tyr66 hydroxyl. An increase in the distance between the His148 side chain and the Tyr66 hydroxyl from 2.89 Å to 3.52 Å indicated the disruption of another H-bond, typically stabilizing the position of the chromophore. These rearrangements in the nearest chromophore environment did not affect its absorbance and emission spectra but greatly impacted its FL, which predominantly depends on the chromophore’s local microenvironment.

### 2.3. Electric and Dynamic Properties of the Chromophore-Binding Site

We assumed that a significant 3.5-fold decrease in the BrUSLEE fluorescence lifetime compared to the EGFP could be a consequence of the changes in the structural, electrostatic, and dynamic properties of the chromophore nearest environment. To explore this issue in detail, and to get further insight into the molecular origin of the observed differences in the photophysical properties of these proteins, we performed all-atom molecular-dynamics (MD) simulations of BrUSLEE and EGFP in water. The results of the MD revealed that, in both FPs, the chromophore preserves well the initial conformation observed in the crystal structures. In particular, its two-pi-electron systems almost do not change their mutual orientation—the angles between the planes of phenolic and imidazolinone rings remain at 7° for both chromophores.

**Figure 4:**
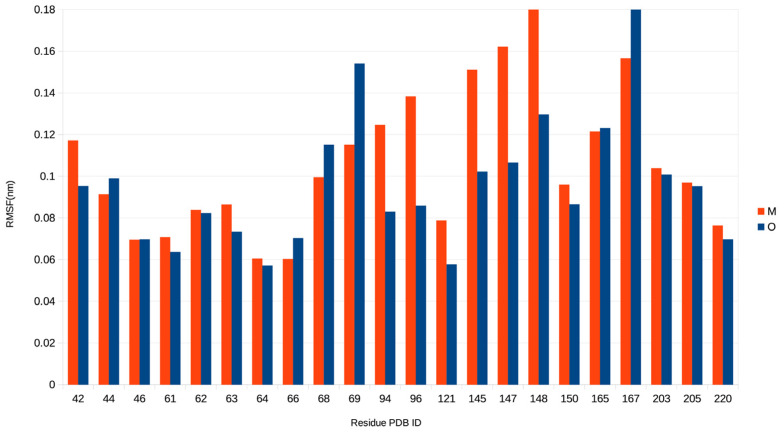
Results of MD simulations; mobility diagram spanning the chromophore region in EGFP (dark blue) and BrUSLEE (red), represented by their respective RMSF values.

On the other hand, the MD data clearly demonstrate increased flexibility of the side chains around the chromophore (within 5 Å) in BrUSLEE compared to the EGFP precursor, thus indicating their greater conformational freedom in the mutant. To evaluate the mobility of the residues forming the nearest chromophore environment, we calculated the values of their root-mean-square fluctuation (RMSF) at the equilibrium part of the MD trajectories (Figure 4). The RMSF values for the residues in both structures show significant differences.

As seen in Figure 4, the mobility of residues Leu42, Gln94, Glu95, Arg96, Asn121, Met145, Ser147, and His148 (see Figure 1) around the chromophore is noticeably higher in BrUSLEE than in EGFP (the reverse picture is observed only for Gln69 and Ile167). Thus, in general, the conformational flexibility of residues in the chromophore-binding site of EGFP is strongly limited. To elucidate the nature of this effect for both proteins, we performed a detailed analysis of interactions between the chromophore and its nearest amino-acid environment at the later stages of MD trajectories. It revealed that EGFP has a significantly higher average number of the protein–chromophore H-bonds and π–π stacking interactions per unit MD time (Table 3).

Compared to BrUSLEE, the parental EGFP chromophore forms ~1.5–2 times more H-bonds with the protein environment, and these bonds are characterized by a greater lifetime. The values of the probability of occurrence of the H-bonds and π–π interactions between the chromophore and its nearest protein environment in EGFP and BrUSLEE are listed in Table 4 (see Figure 1, Figure 2 and Figure 3). The Gln94, Arg96, and His148 in BrUSLEE demonstrate the highest variation of RMSF values relative to the parental EGFP precursor (Figure 4).

**Figure 5:**
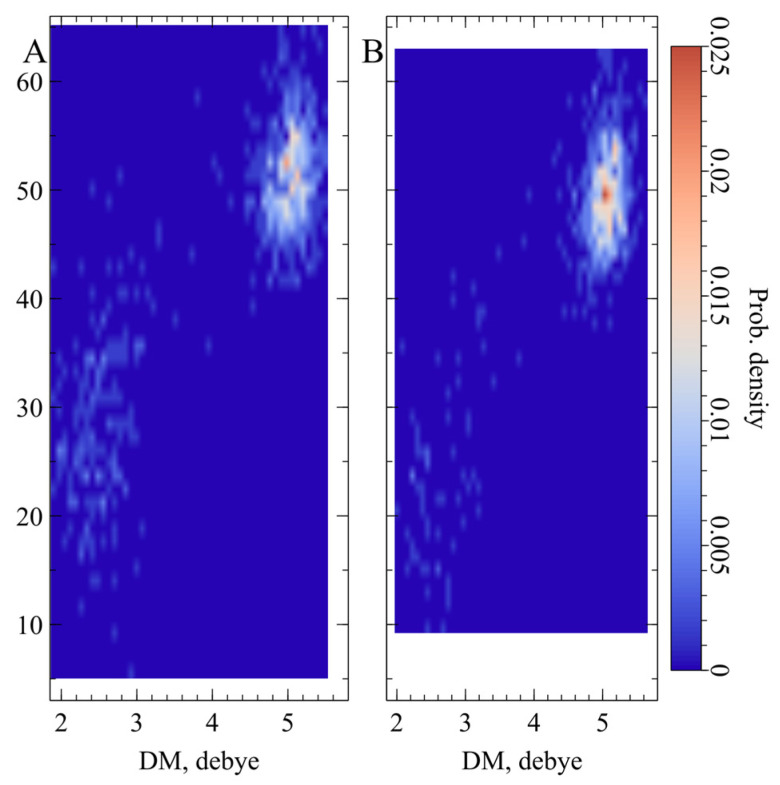
MD-derived two-dimensional distribution of the modulus of the chromophore dipole moment (DM) (*X*-axis) vs. the angle between the CG2-CZ vector in the phenolic ring of the chromophore and the DM vector (*Y*-axis) in the EGFP (**A**) and BrUSLEE (**B**). The values of the probability density of the occurrence of MD states are given according to the color scale (on the right).

Since the photophysical properties of the studied protein directly depend on the electric field in the chromophore area, we also used MD simulations to get the relevant information. As a result, we delineated two main electronic states in EGFP and BrUSLEE, differing in the orientation of the dipole moment (DM) vector of the chromophore (Figure 5). The most populated state is characterized by the DM of ~5.0 ± 0.3 D with an orientation of ~50° to the CG2-CZ(OH) axis of the chromophore phenolic ring. The minor state is much more diffuse, having a DM of ~2.5 ± 0.4 D with a corresponding orientation of ~25°. These states correspond to different orientations of the O-H and C=O groups of the chromophore phenolic and imidazolinone rings. The hydroxyl proton has either a trans- (major state) or cis- (minor state) configuration with respect to the oxygen in the carbonyl group.

### 2.4. Conclusive Summary

The green-fluorescent biomarker BrUSLEE, characterized by a short fluorescent lifetime (FL) of ~0.66 ns has been studied by X-ray and molecular dynamics methods. The protein demonstrates an exceptional fluorescence brightness, reaching ~78% of that of the green EGFP precursor (FL~2.83 ns). BrUSLEE allows the recording of a distinct FL signal in the presence of spectrally similar GFPs in multiparameter FLIM experiments. Its development from the EGFP precursor by three mutations in the chromophore area (Thr65Gly, Tyr145Met, and Phe165Tyr) resulted in a change of the chromophore microenvironment due to a significant rearrangement in the side chain conformations.

The mutations increased the free volume around the chromophore, causing a shift of the Tyr66 hydroxyl group of the chromophore by 0.7 Å. The following increase in the conformational freedom of the residues in the chromophore area was confirmed by a molecular dynamics (MD) simulation. Compared to the EGFP precursor, which is characterized by an increased packing density in the chromophore area, in BrUSLEE the stabilizing contacts of the chromophore with the environment were noticeably weaker. The average number of protein–chromophore H-bonds and stacking interactions was 1.5 times less (Table 3 and Table 4). This, in turn, resulted in higher mobility of its neighboring residues; for some of them, the increase in the corresponding RMSF values reached 40%, (Figure 4). The aforementioned reorganization of the chromophore-binding site in BrUSLEE as compared to its precursor also changed the electric properties of the chromophore and its microenvironment. This significantly shifted the populations of the two main electronic states with different dipole moments observed in the course of MD simulations. The major state was characterized by DM~5.0 D with a ~50° orientation to the CG2-CZ(OH) axis of the chromophore phenolic ring, while the more diffuse minor state had a DM~2.5 D with a corresponding ~25° orientation.

## 3. Materials and Methods

### 3.1. Crystallization

BrUSLEE was dialyzed against 20 mM Tris pH 8.0 200 mM NaCl buffer and concentrated to 40 mg/mL. Crystals suitable for data collection were obtained from 6.4% Tacsimate pH 5.0, 16% PEG 3350 by hanging drop experiment in which 2 μL of the protein were mixed with 2 μL of the reservoir solution and incubated against the same reservoir at 20 °C for two weeks. The chemicals for crystallization were obtained from Hampton Research (Aliso Viego, CA, USA).

### 3.2. X-ray Data Collection, Structure Solution, and Crystallographic Refinement

The X-ray experiment was collected at the SER-CAT BM line at APS. Prior to data collection, the crystal was briefly dipped into the cryoprotecting solution containing the reservoir solution plus 30% glycerol and flash frozen in a 100 K nitrogen stream. All diffraction images were processed with HKL2000 [13]. Crystal structures were solved by the molecular replacement method with MOLREP [14,15] using the coordinates of EGFP (PDB ID: 4EUL) as a search model. Crystallographic refinement was performed with REFMAC5 [16], alternating with manual revision of the model with COOT [17]. Crystallographic data and refinement statistics are given in Table 5. The coordinates of BrUSLEE were deposited in the Protein Data Bank under accession code PDB_ID: 8BVG.

### 3.3. Molecular Dynamics Simulation

The starting models of EGFP and BrUSLEE, corresponding to their crystal structures, were taken from the Protein Data Bank (PDB entries 2Y0G and 8BVG, respectively). Both proteins were placed in rhombic dodecahedron boxes (A = B = C = 100 Å, α = β = γ = 60°) and solvated with explicit TIP3P water [18]. Na^+^ ions [19] were added to neutralize the total charge of the system. Molecular dynamics (MD) simulations were carried out with the GROMACS software package version 2021.5 [20] using the CHARMM36 force field [21]. An integration time step of 2 fs was used, and 3D periodic boundary conditions were imposed. Simulations were performed with a constant temperature (325 K) and pressure (1 bar) maintained using the V-rescale [22] and the Parrinello–Rahman [23] algorithms, respectively. The isotropic pressure coupling was used in the simulations. The 12 Å cutoff radius was defined for the Coulombic and van der Waals interactions. Electrostatic interactions were evaluated using the particle-mesh Ewald (PME) summation [24] (real-space cutoff of 12 Å). Protein and solvent molecules were coupled separately. Partial charges and bonded parameters of the chromatophore were obtained from Reuter et al. [25]. Missing values were transferred from the protein [21] or CGenFF [26] CHARMM36 parameter sets by analogy.

The simulated systems were first equilibrated in several stages: 3000 steps of steepest descent minimization followed by heating from 5 K to 325 K during a 100-ps MD run, in which coordinates of the protein heavy atoms were restrained to permit solvent relaxation. In the next step, the systems were subjected to 50 ns of MD equilibration with fixed protein Cα atoms. Then, 100 ns long production-MD simulations without restraints were performed. Atomic coordinates from the MD trajectories were analysed with a timestep of 100 ps using original GROMACS and in-house utilities. Lastly, 75 ns of production MD run were used in the analysis. The per-residue values of the root-mean-square fluctuations (RMSF) were calculated using the rmsf utility from the GROMACS package. The dipole moment (DM) of the chromatophore part (imidazolone ring, aryl-alkene bond, and phenol ring) was calculated using the CHARMM36 force field partial charges. The intermolecular contacts (including H-bonds), DM parameters, and the geometrical characteristics of the chromophore were calculated using in-house software.

## Figures and Tables

**Table 1:** Spectral properties of BrUSLEE [10].

Fluorescent Protein	λex/λem	EC, M^−1^·cm^−1^	FQY	Relative Brightness *, %	FL, ns	Relative Photostability, %	
						In Vitro	In Cellulo
EGFP	489/509	55,000	0.60	100	2.83 ± 20	100 ± 8	100 ± 20
BrUSLEE	487/509	86,000	0.30	78	0.66 ± 35	190 ± 4	230 ± 45

* Relative brightness (compared to EGFP) is the product of molar extinction coefficient (EC) and fluorescence quantum yield (FQY).

**Table 2:** H-bonds and hydrophobic interactions at the E-F dimer interface.

H-Bonds *	d (Å)
Asn146(ND2)∙∙∙∙(OD1)Asn146	3.28
Asn(144)(ND2)∙∙∙∙(OG)Ser147	3.17
Asn(170)(ND2)∙∙∙∙(OG) Ser147	3.59
Leu207(O)∙∙∙∙(NE2)Gln204	2.66
Asn212(ND2)∙∙∙∙(OH)Tyr39	3.62
Hydrophobic cluster *	
Phe123 + Leu221 + Ala206	

* The numbers of four H-bonds and the residues in the hydrophobic cluster are doubled due to a 2-fold crystallography symmetry axis.

**Table 3:** Average number of protein–chromophore interactions per unit of MD time.

Chromophore	H-Bonds	π–π
EGFP	3.6	0.81
BrUSLEE	2.4	0.37

**Table 4:** Probability of occurrence (PrOc) of H-bonds and π–π interactions in the chromophore areas.

H-Bonds			
EGFP		BrUSLEE	
Residue	PrOc	Residue	PrOc
VAL61	0.98	VAL61	0.92
ARG96	0.83	ARG96	0.49
GLN94	0.77	GLN94	0.33
HIS148	0.5	SER205	0.25
GLN69	0.23	GLN69	0.22
TYR145	0.09	SER147	0.16
**π–π Interactions**			
PHE165	0.81	TYR165	0.37

**Table 5:** Crystallographic data and refinement statistics.

Protein	BrUSLEE
**Crystallographic data**	
Space group	P2_1_2_1_2_1_
Cell dimensions (Å, °)	a = 75.66, b = 122.71 c = 167.40
Z(Z’).	4(6)
Estimated solvent content (%)	47.0
Temperature (K)	100
Wavelength (Å)	1.00
Resolution range (Å)	29.72–2.38 (2.49–2.38)
Total observations	427,530
Unique reflections observed	62,234
Redundancy	6.9 (5.6)
*I/σ(I)*	20.1 (1.6)
*R_merge_*	0.094 (0.930)
Completeness (%)	99.9 (99.3)
**Refinement statistics**	
Non-H atoms in model	
Protein	1826 [24 × 231 res)]
Water	185
*R_work_*	0.203
*R_free_*	0.271
Fraction of free reflections (%)	2.0
RMSD from ideal values:	
Bond lengths (Å)	0.011
Bond angles (grad)	1.404
Torsion angles (period 3; grad)	17.2
Chirality (Å^3^)	0.074
General planes (Å)	0.014
**Ramachandran statistics (%)**	
Preferred/Allowed/Outliers	95.8/4.0/0.2

## Data Availability

Not applicable.

## References

[1] Chudakov D.M., Matz M.V., Lukyanov S., Lukyanov K.A. (2010). Fluorescent Proteins and Their Applications in Imaging Living Cells and Tissues. Physiol. Rev..

[2] Wu B., Piatkevich K.D., Lionnet T., Singer R.H. (2011). Modern Fluorescent Proteins and Imaging Technologies to Study Gene Expression, Nuclear Localization, and Dynamics. Curr. Opin. Cell Biol..

[3] Lam A.J., St-Pierre F., Gong Y., Marshall J.D., Cranfill P.J., Baird M.A., McKeown M.R., Wiedenmann J., Davidson M.W., Schnitzer M.J. (2012). Improving FRET dynamic range with bright green and red fluorescent proteins. Nat. Methods.

[4] Mishin A.S., Belousov V.V., Solntsev K.M., Lukyanov K.A. (2015). Novel uses of fluorescent proteins. Curr. Opin. Chem. Biol..

[5] Cardarelli F. (2020). Back to the Future: Genetically Encoded Fluorescent Proteins as Inert Tracers of the Intracellular Environment. Int. J. Mol. Sci..

[6] Periasamy A., Clegg R.M. (2020). FLIM Microscopy in Biology and Medicine.

[7] Datta R., Heaster T.M., Sharick J.T., Gillette A.A., Skala M.C. (2020). Fluorescence lifetime imaging microscopy: Fundamentals and advances in instrumentation, analysis, and applications. J. Biomed Opt..

[8] Margineanu A., Chan J.J., Kelly D.J., Warren S.C., Flatters D., Kumar S., Katan M., Dunsby C.W., French P.M.W. (2016). Screening for protein-protein interactions using Förster resonance energy transfer (FRET) and fluorescence lifetime imaging microscopy (FLIM). Sci. Rep..

[9] Mamontova A.V., Solovyev I.D., Savitsky A.P., Shakhov A.M., Lukyanov K.A., Bogdanov A.M. (2018). Bright GFP with subnanosecond fluorescence lifetime. Sci. Rep..

[10] Mamontova A.V., Shakhov A.M., Lukyanov K.A., Bogdanov A.M. (2020). Deciphering the Role of Positions 145 and 165 in Fluorescence Lifetime Shortening in the EGFP Variants. Biomolecules.

[11] Wallace A.C., Laskowski R.A., Thornton J.M. (1995). LIGPLOT: A program to generate schematic diagrams of protein-ligand interactions. Protein Eng..

[12] DeLano W.L. (2002). The PyMOL Molecular Graphics System.

[13] Otwinowski Z., Minor W. (1997). Processing of X-ray diffraction data collected in oscillation mode. Methods Enzymol..

[14] Vagin A., Teplyakov A. (2010). Molecular replacement with MOLREP. Acta Cryst. D Biol. Cryst..

[15] Winn M.D., Ballard C.C., Cowtan K.D., Dodson E.J., Emsley P., Evans P.R., Keegan R.M., Krissinel E.B., Leslie A.G., McCoy A. (2011). Overview of the CCP4 suite and current developments. Acta Cryst. D Biol. Cryst..

[16] Murshudov G.N., Skubak P., Lebedev A.A., Pannu N.S., Steiner R.A., Nicholls R.A., Winn M.D., Long F., Vagin A.A. (2011). REFMAC5 for the refinement of macromolecular crystal structures. Acta Cryst. D Biol. Cryst..

[17] Emsley P., Lohkamp B., Scott W.G., Cowtan K. (2010). Features and development of Coot. Acta Cryst. Biol. Cryst..

[18] Jorgensen W.L., Chandrasekhar J., Madura J.D. (1983). Comparison of simple potential functions for simulating liquid water. J. Chem. Phys..

[19] Beglov D., Roux B. (1994). Finite representation of an infinite bulk system: Solvent boundary potential for computer simulations. J. Chem. Phys..

[20] Abraham M.J., Murtola T., Schulz R., Pall S., Smith J.C., Hess B., Lindahl E. (2015). GROMACS: High performance molecular simulations through multi-level parallelism from laptops to supercomputer. SoftwareX.

[21] MacKerell A.D., Bashford D., Bellott M.L., Dunbrack R.L., Evanseck J.D., Field M.J., Fischer S., Gao J., Guo H., Ha S. (1998). All-Atom Empirical Potential for Molecular Modeling and Dynamics Studies of Proteins. J. Phys. Chem. B.

[22] Bussi G., Donadio D., Parrinello M. (2007). Canonical sampling through velocity rescaling. J. Chem. Phys..

[23] Parrinello M., Rahman A. (1981). Polymorphic transitions in single crystals: A new molecular dynamics method. J. Appl. Phys..

[24] Essmann U., Perera L., Berkowits M.L., Darden T., Lee H., Pedersen L.G. (1995). A smooth particle mesh Ewald method. J. Chem. Phys..

[25] Reuter N., Lin H., Thiel W. (2002). Green Fluorescent Proteins:  Empirical Force Field for the Neutral and Deprotonated Forms of the Chromophore. Molecular Dynamics Simulations of the Wild Type and S65T Mutant. J. Phys. Chem. B.

[26] Vanommeslaeghe K., Hatcher E., Acharya C., Kundu S., Zhong S., Shim J., Darian E., Guvench O., Lopes P., Vorobyov I. (2010). CHARMM general force field: A force field for drug-like molecules compatible with the CHARMM all-atom additive biological force fields. J. Comput. Chem..

